# Screening for anti-inflammatory components from *Corydalis bungeana* Turcz. based on macrophage binding combined with HPLC

**DOI:** 10.1186/s12906-015-0907-x

**Published:** 2015-10-15

**Authors:** Zi-Bo Dong, Yong-Hong Zhang, Bing-Jie Zhao, Chao Li, Gang Tian, Ben Niu, Hong Qi, Liang Feng, Jian-Guo Shao

**Affiliations:** Post-doctoral Research Center, Nanjing University of Chinese Medicine & Jumpcan Pharmaceuticl Co.,Ltd, Jiangsu Taizhou, 225441 P. R. China; Nanjing University of Chinese Medicine, Jiangsu Nanjing, 210046 P. R. China; Jumpcan Pharmaceuticl Co., Ltd, Jiangsu Taizhou, 225441 P. R. China

**Keywords:** *Corydalis bungeana* Turcz., Anti-inflammatory, Macrophage, HPLC

## Abstract

**Background:**

Corydalis bungeana Turcz. (CB; family: Corydalis DC.) is an anti-inflammatory medicinal herb used widely in traditional Chinese medicine (TCM) for upper respiratory tract infection, etc., but its anti-inflammatory active molecules are unknown. This study was designed to screen for the anti-inflammatory components from CB based on macrophage binding combined with HPLC.

**Methods:**

Xylene-induced ear edema in mouse and carrageenan-induced hind-paw edema in rats were used to evaluate the anti-inflammatory activity of CB. The macrophage binding with high-performance liquid chromatography (HPLC) analysis and HPLC-MS were established to screen the potential active compounds. ELISA kits were performed to measure the levels of IL-6, IL-10, TNF-α and NO in RAW 264.7 macrophages culture media.

**Results:**

The alkaloid extract of CB could inhibit significantly xylene-induced ear edema in mouse and carrageenan-induced hind-paw edema in rats. Two components binded to RAW 264.7 cell were identified as 12-hydroxycorynoline and corynoline. Bioassays demonstrated that these two compounds significantly inhibited LPS-induced IL-6, IL-10, TNF-α and NO levels.

**Conclusions:**

The results suggest that corynoline and 12-hydroxycorynoline contribute to the anti-inflammatory effects of the alkaloid extract of CB. Our findings suggest that these two compounds can be used as candidate for anti-inflammatory drugs.

## Background

*Corydalis bungeana* Turcz. (CB; family: Corydalis) is a perennial herb containing several pharmacologically important alkaloids such as corydaline, 12-hydroxycorynoline, protopine, acetylcorynoline, and corynoline [1]. The dried whole plant is referred in traditional Chinese medicine (TCM) as Herba Corydalis Bungeanae, clearing heat and toxins, as well as anti-inflammatory [2]. Corynoline is the major alkaloid component derived from CB. It has been known that corynoline, acetylcorynoline, and protopine can significantly reduce carbon tetrachloride (CCl_4_)-induced microsomal lipid peroxidation [3] and attenuate two, 4-dinitro-1-fluorobenzene-induced delayed-type hypersensitivity [2]. Some isoquinoline alkaloids such as 12-hydroxycorynoline isolated from CB have bacteriostatic activity [4]. CB has been used for treating influenza, infections of the upper respiratory tract, bronchitis, tonsillitis, acute nephritis, and pyelonephritis [5]. However, the molecules responsible for treating inflammatory diseases have not been identified.

Extraction and purification of compounds from herbs is time-consuming. Screening of bioactive compounds for use in animal models of disease is also laborious and inappropriate for TCMs [6]. The action of drugs is usually demonstrated a fact that the drug molecules interacted with receptors, enzymes or channels on cell membranes, and then mediated signaling cascade [7]. A screening method involving in the binding of molecules to the membranes of human red blood cells and high-performance liquid chromatography (HPLC) analysis was developed to identify the active components of herb extract [8]. Here, we report an improved method based on macrophage binding and HPLC. Cell suspensions were treated with the alkaloid extract of CB and then centrifuged to remove unbound components. The final eluate was collected as a sample for screening potential active components in medicinal herbs by HPLC analysis.

Lipopolysaccharide (LPS) is an endotoxin and a constituent of the outer membrane of Gram-negative bacteria [9]. LPS-stimulated macrophages can produce various inflammatory mediators: interleukins (such as IL-6, IL-8, etc.), interferon (IFN)-γ and TNF-α [10]. Levels of inflammatory mediators in the supernatants of LPS-stimulated macrophages are reliable markers of inflammation. Various chemicals in plants show anti-inflammatory activity by modulating levels of inflammation-associated genes and inhibiting the release of inflammation-associated mediators. Hence, stimulation of macrophages with LPS offers an excellent model for screening and subsequent evaluation of the inflammatory effects of candidate drugs.

In this study, we screened the components from the extract of CB and then evaluated the effects of components screened from the alkaloid extract of CB using LPS-stimulated RAW 264.7 cells (a macrophage cell line) by examining cytokine production.

## Methods

### Plant materials and reagents

The samples of CB were purchased from Anhui Pharmaceutical Company (An’hui, China; voucher specimen ID number: 20120425). Botanic identification was confirmed by Professor Chungen Wang (Nanjing University of Chinese Medicine, Nanjing, China). Specimens are stored in the Herbal Specimens Center of Jumpcan Pharmaceuticl Co.,Ltd (Stored specimen No. JC-2012-005). Protopine, 12-hydroxycorynoline, 6-acetonylcory-noline, corynoline and acetylcorynoline were prepared by the Nanjing University of Chinese Medicine and purity was 99 % according to HPLC analysis. Aspirin was obtained from Nanjing Baijingyu Pharmaceutical Co., Ltd. (Nanjing, China). Xylene was purchased from Shanghai Qiangshun Pharmaceutical Co., Ltd. (Shanghai, China). LPS and carrageenan were obtained from Sigma–Aldrich (Saint Louis, MO, USA). Dulbecco’s modified Eagle’s medium (DMEM) was purchased from Gibco (Grand Island, NY, USA). Fetal bovine serum (FBS) was obtained from Wisent Biological Corporation (Nanjing, China). Enzyme-linked immunosorbent assay (ELISA) kits for determination of the levels of IL-6, IL-10 and TNF-α were purchased from eBioscience (San Diego, CA, USA). Kits for the measurement of nitric oxide (NO) was obtained from Beyotime Institute of Biotechnology (Shanghai, China). The murine macrophage cell line (RAW 264.7 cells) was kindly provided by Professor Xu Qiang (Nanjing University, Nanjing, China). Water for HPLC analysis was purified using a Milli-Q Water Purification system (Millipore, Bedford, MA, USA). Other reagents were of analytical grade.

### Animals

ICR mice (18–24 g) and Sprague–Dawley (SD) rats (150–200 g) were purchased from the Zhejiang Experimental Animal Center (Zhejiang, China). All animals were maintained at the Nanjing University of Chinese Medicine under specific pathogen-free conditions. The study protocol was authorized by the Ethics Committee of Nanjing University of Chinese Medicine (Nanging, China) in accordance with the Guide for the Care and Use of Laboratory Animals (National Institutes of Health, Bethesda, MD, USA).

### Extraction and isolation of alkaloids

Air-dried herbs (20 kg) were extracted twice with 200 and 160 l of 60 % (v/v) ethanol (blend 95 % (v/v) ethanol with appropriate water to 60 %) for 1 h successively. Extracts were collected and concentrated under reduced pressure at 55 °C. Concentrates were suspended in water, adding 1 mol/L HCl to adjust pH 2 of the solution, left for 4 h, then 40 % (v/v) NaOH (40 g NaOH dissolved in 100 ml of water) mixed with the liquor until its pH 9, left overnight. Extracts (300 g) were filtered to obtain a fraction of crude alkaloids and then extracted with a ten-fold volume of 95 % (v/v) ethanol under reflux for 1 h. This extraction was repeated twice. The extract was collected and concentrated under reduced pressure at 55 °C (i.e., 20 g of alkaloid components with minimum polarity). The residue was extracted with equal volumes of butanol thrice and combined with butanol. The extract was collected and concentrated under reduced pressure at 55 °C (i.e., alkaloid components with maximum polarity). The samples (i.e., alkaloid extract of CB) were ready for HPLC analysis.

### Cell culture and binding

RAW 264.7 macrophages were cultured in DMEM supplemented with 10 % FBS, antibiotics (10 U/mL penicillin G, 100 g/mL streptomycin), and maintained at 37 °C in a humidified incubator containing 5 % CO_2_. Cell suspensions (10 mL; 2 × 10^7^ cells) were treated with the alkaloid extract of CB (0.5 μg/mL) for 1 h at 37 °C. Suspensions were then centrifuged for 15 min at 1000 rpm (175 × g) at room temperature and cell pellets were washed for ten times to remove unbound components. Washed eluates were discarded except for the final eluate, which was collected as a control for HPLC analysis (sample A). RAW 264.7 macrophages were denatured by addition of 10 mL hydrochloric acid and phosphate-buffered saline (PBS; pH 4.0) to liberate components bound to cells whithout damaging the physiological state of cells. The desorption eluate was clarified by centrifugation and retained for HPLC analysis (sample B). Cells treated with dimethyl sulfoxide (DMSO) alone were used as the vehicle control of the alkaloid extract of CB, as the vehicle desorption eluate (sample C). Aliquots (1 mL) of samples A, B and C were mixed separately with 1 mL methanol, vortex-mixed for 2 min, and centrifuged at 10,000 rpm for 3 min at room temperature. Supernatants were collected and dried under nitrogen at 45 °C. Residues were taken up into 200 μL methanol and filtered through a 0.45-μm nylon membrane before HPLC analysis.

### HPLC analysis

The alkaloid extract of CB and RAW 264.7 macrophage-bound samples were analyzed by HPLC. We used a Series 1100 Liquid Chromatograph (Agilent Technologies, Palo Alto, CA, USA) equipped with a vacuum degasser, a quaternary pump, an autosampler and a photodiode-array detector (PDA) connected to Agilent ChemStation software. A phenomenex Gemini C18 ODS column (4.6 mm × 250 mm, 5 μm) was used. The mobile phase was (A) ammonium acetate (10 mM; pH 9.5); (B) methanol. The flow rate was 1 mL/min. The elution conditions were: B, 0–45 min, linear gradient 15–60 % B; 45–70 min, linear gradient 60–86 % B; 70–80 min, linear gradient 86–15 % B. The system operated at 30 °C and the injection volume was 10 μL. The detection wavelength was kept at 289 nm.

### HPLC-mass spectrometry (MS) analysis

MS conditions were: positive ion model; drying gas, N_2_; spray tip potential, 5290 V; nozzle potential, 140 V; nozzle temperature, 140 °C; detector voltage, 2100 V; scan range, 50–1000 m/z. An Alliance 2690 HPLC instrument (Waters, Milford MA, USA) coupled with a UV detector was used for quantitative determination. The chromatographic conditions were as described above. Chromatograms were monitored at 289 nm. The structure was confirmed by H-NMR and C^13^-NMR. (5 mg sample was dissolved in d6-CDCl_3_, and detected by AV-500. BRUKER.)

### Evaluation of anti-inflammatory activity in vitro

RAW 264.7 macrophages (1.2 × 10^6^) were cultured in DMEM supplemented with 10 % FBS and maintained at 37 °C in a humidified incubator containing 5 % CO_2_. Cells were stimulated with LPS (0.5 μg/mL) for 24 h and treated with different concentrations (100 μg/mL, 10 μg/mL, 1 μg/mL, 100 ng/mL and 10 ng/mL) of compounds obtained from the alkaloid extract of CB for 1 h. Control values were obtained in the absence of LPS and compounds. Levels of IL-6, IL-1β, NO and TNF-α in macrophage culture media were measured by ELISA kits according to manufacturer’s instructions.

### Xylene-induced ear edema in mice

The xylene-induced ear edema test with modifications was conducted as described previously [11]. Briefly, four groups of ten mice were treated (via the oral route) with physiological (0.9 %) saline (control), alkaloid extract of CB (40 and 80 mg/kg) or aspirin (150 mg/kg; positive control). Mice were co-administrated (by gavage) with different doses of the alkaloid extract of CB intragastrically once a day for 5 days. About 1 h after administration, each mouse received 50 μL of xylene on the inner and outer surfaces of the right ear. A further 1-h later, ear thickness was measured with a Thickness Gauge (Harbin Measuring & Cutting Tool Group Co., Ltd., Harbin, China). Mice were killed by cervical dislocation. Ear biopsies (diameter, 9.0 mm) were punched out and weighed. The degree of ear swelling was calculated based on the weight difference between the biopsies of the right and left ear of the same mouse. Percent inhibition of edema was calculated using the formula:

$$ \mathrm{Edema}\ \mathrm{inhibition}\ \left(\%\right) = \left({\mathrm{edema}}_{\mathrm{control}}\hbox{-} \kern0.5em {\mathrm{edema}}_{\mathrm{drugs}}\right)/{\mathrm{edema}}_{\mathrm{control}}\kern0.5em  \times 100\% $$

### Carrageenan-induced paw edema in rats

The method used for this investigation was similar to that reported previously [11]. Forty male SD rats were divided into four groups: model control (saline); alkaloid extract of CB (30 mg/kg and 60 mg/kg); aspirin (400 mg/kg).

The normal volume of the right hind-paw of each rat was measured with a Water Displacement Plethysmometer (YLS-7B; YiyanTechnology Co, Ltd, Jinan, China) after the final administration of extract. Then, each rat was treated with 0.1 mL of 1 % carrageenan by subcutaneous injection into the right hind-paw 1 h after administration. Paw volume was measured again at 0.5, 1 and 3 h after the induction of inflammation. Swelling volume was calculated as V_2_–V_1_, where V_1_ and V_2_ are the right hind-paw volumes (mL) of the first and second measurements, respectively.

### Statistical analysis

Data are expressed as the mean ± SD. Comparisons between groups were analyzed by one-way ANOVA. Dunnett’s test was employed for comparison between two groups. *p* < 0.05 was considered significant.

## Results

### Effect of the alkaloid extract of CB on xylene-induced ear edema in mice

**Fig. 1:**
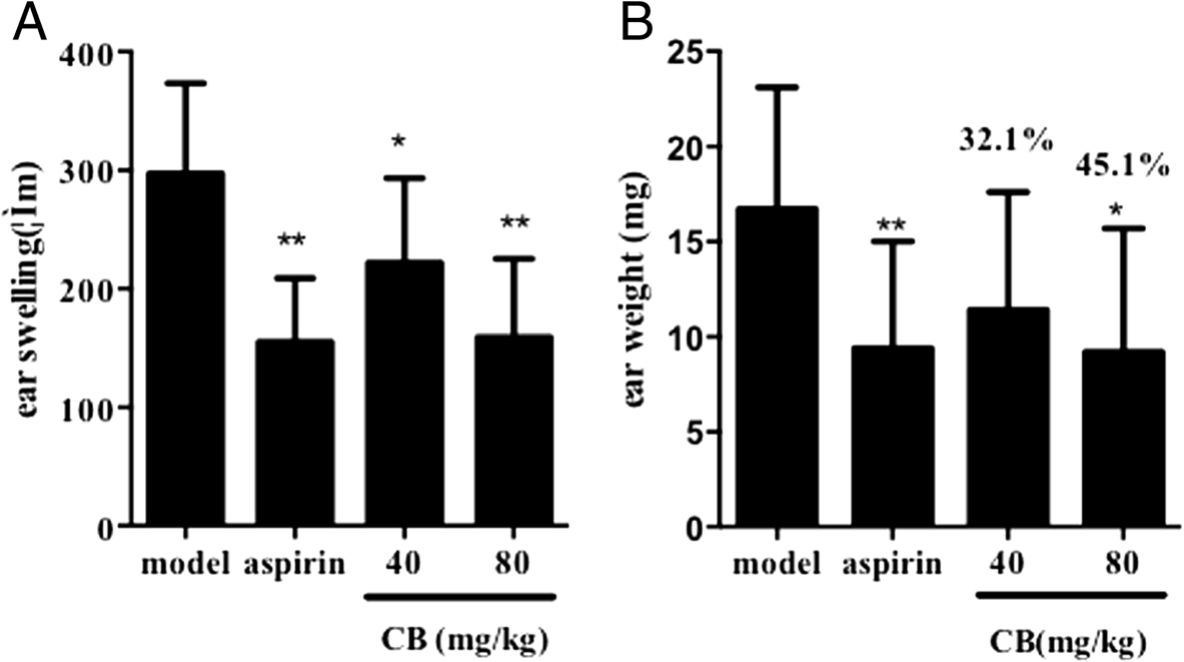
Anti-inflammatory effect of the alkaloid extract of CB on ear swelling (**a**) and ear weight (**b**) in xylene-induced mice. Values are expressed as mean ± SD (*n* = 10). *p < 0.05 and **p < 0.01 vs. model group

Utilizing a typical model of inflammatory response (xylene-induced ear edema), the anti-inflammatory activity of the alkaloid extract of CB was investigated. The alkaloid extract of CB (40 and 80 mg/kg) showed significant inhibition of xylene-induced mice ear edema as compared with the control, with inhibition of 32.1 and 45.1 % (*P* < 0.01) respectively (Fig. 1).

### Effect of the alkaloid extract of CB on carrageenan-induced hind-paw edema

**Fig. 2:**
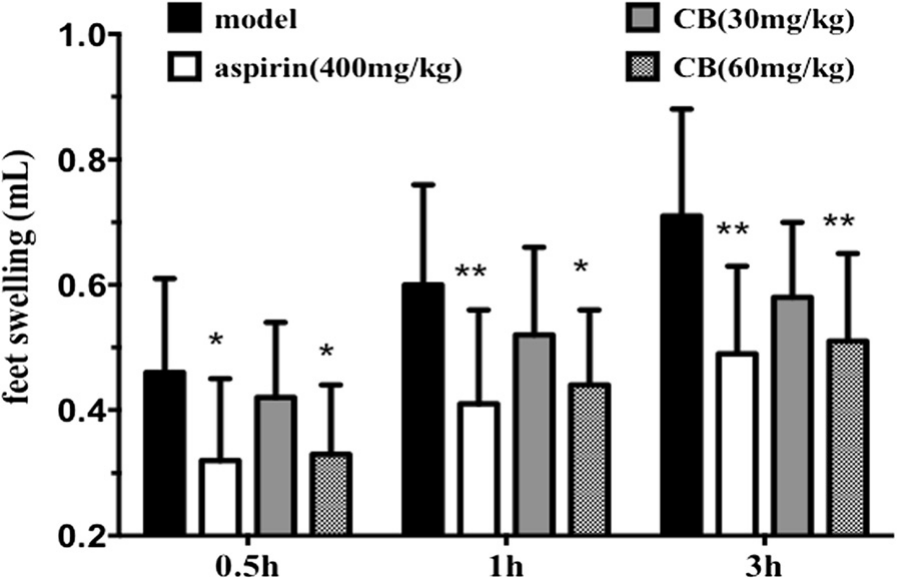
Anti-inflammatory effect of the alkaloid extract of CB on carrageenan-induced hind-paw edema in rats. Values are expressed as mean ± SD (*n* = 10). *p < 0.05 and **p < 0.01 vs. model group

Carrageenan-induced paw inflammation is an acute and highly reproducible model of acute inflammatory response [12]. Cardinal signs of inflammation develop immediately after subcutaneous injection and result from action of pro-inflammatory agents. The inflammatory response is usually quantified by increase in paw size (edema) and is modulated by inhibitors within the inflammatory cascade [13]. A bar chart is given to illustrate the anti-inflammation effect of the alkaloid extract of CB in Fig. 2. The results indicate that the swelling volume increased progressively and peaked at 3rd hour after carrageenin injection. However, the edema of rat paw was significantly reduced by the treatment with the high-dose alkaloid extract of CB (60 mg/kg) during all phases of carrageenan-induced inflammation. And the inhibition ratios of the CB (60 mg/kg) treatment with rats at 0.5, 1 and 3 h were 29.8 % (*P <* 0.05), 28.3 % (*P* < 0.05) and 28.6 % (*P <* 0.01), respectively, compared to the model control. Aspirin was used as a positive control and had similar effects (31.9, 32.5 and 30.0 % inhibition, respectively).

### HPLC analysis for the alkaloid extract of CB

**Fig. 3:**
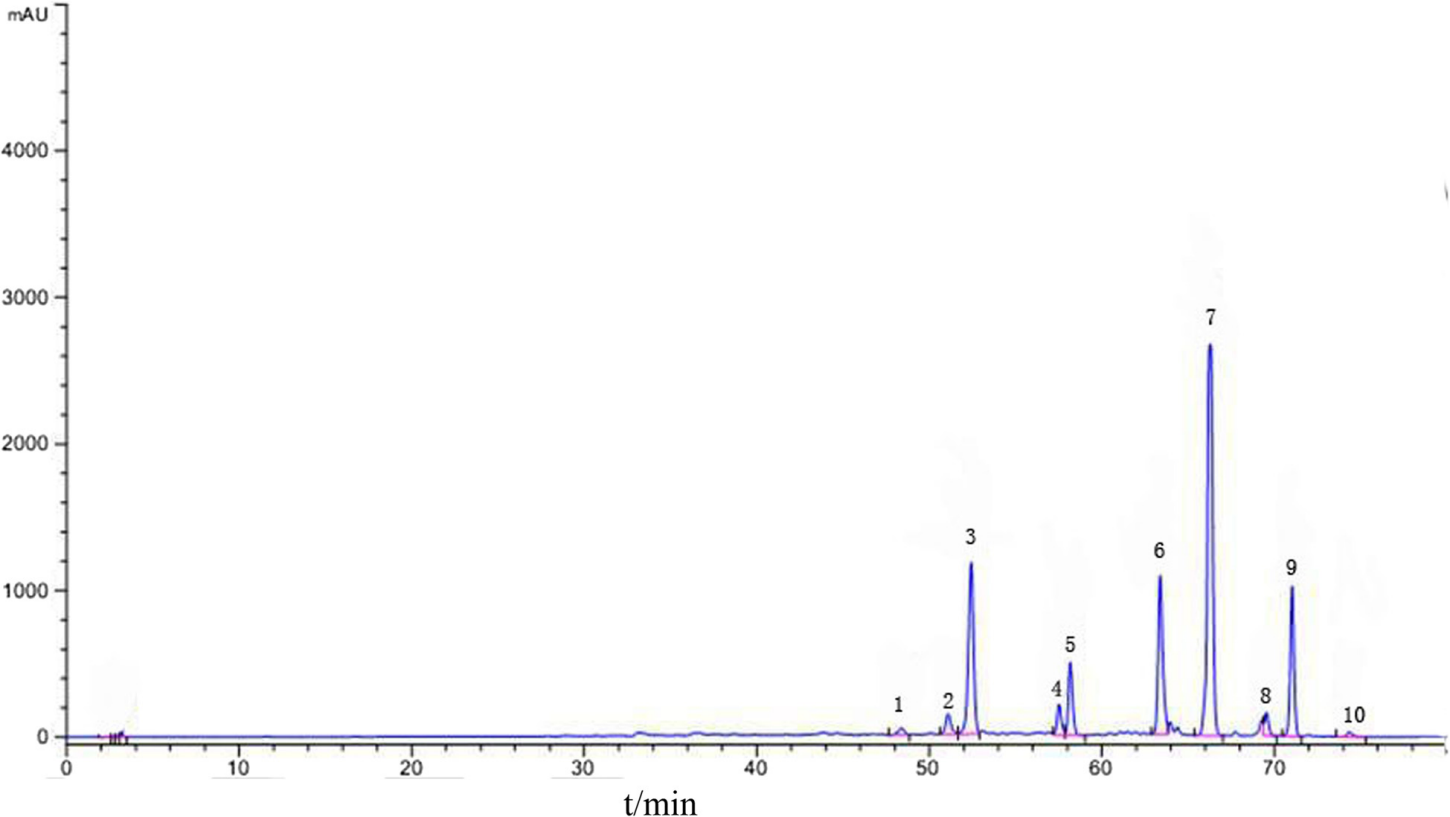
HPLC fingerprint of the alkaloid extract of *Corydalis bungeana* Turcz

First, we established the fingerprint of the alkaloid extract of CB as the background information of macrophage binding. There were ten main peaks in the fingerprint of the alkaloid extract of CB at 289 nm (Fig. 3). Among these, five components were determined after comparison with standards (Table 1). All of the main peaks had good separation.

**Table 1:** RRT and the RPA of each peak in the fingerprint of the alkaloid extract of CB

Peak number	RRT	RPA	Identified compound
1	1.370	47.444	unknown
2	1.297	19.862	unknown
3	1.264	2.367	Protopine
4	1.152	15.868	unknown
5	1.139	6.923	12-hydmxycorynoline
6	1.046	2.849	6-acetonylcorynoline
7	1.000	1.000	Corynoline
8	0.953	21.822	Acetylcorynoline
9	0.933	3.622	unknown
10	0.892	75.680	unknown

Injection precision was determined by five replicate injections of the same sample in one day. The relative standard deviations (RSDs) of relative retention time (RRT) and relative peak area (RPA) were 0.72 and 0.56 %, respectively, and the changes were not significant. Repeatability was assessed by analyzing five independently prepared samples of the alkaloid extract of CB. RSDs of RRT and RPA were 1.22, 1.57 %, respectively, and the changes were not significant. Sample stability was assessed by successive injections of the same sample at 0, 1, 2, 4, 8, 12 and 24 h. During this period, the solution was stored at room temperature. RSDs of RRT and RPA were 1.72 and 2.05 %, respectively, and the changes were not significant. Results of injection precision, repeatability and the stability test suggested that this method was adequate, valid and applicable. RRT and RPA of each characteristic principle was calculated using corynoline as the reference peak. The RRT and RPA of the ten peaks are shown in Table 1.

### Binding of the alkaloid extract of CB to RAW 264.7 cells

The alkaloid extract of CB exerted anti-inflammatory properties, so we used its binding to RAW 264.7 cells to screen the active compounds within it. The alkaloid extract of CB was incubated with RAW 264.7 cells and bound components collected by centrifugation. After thoroughly washing and eluting from cell pellets, retained components were injected into HPLC system for analysis.

**Fig. 4:**
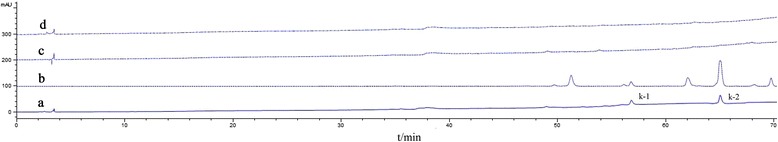
Detection of RAW 264.7 cells-binding molecules in the alkaloid extract of CB by HPLC analysis. **a** Comparative chromatograms of the desorption eluate of alkaloid extract of CB. **b** the fingerprint of the alkaloid extract of CB. **c** the final wash eluate and **d** the vehicle desorption eluate. Detection was at 289 nm

Two principal peaks (K-1 and K-2) were detected in sample B (desorption eluate of the alkaloid extract of CB) (Fig. 4a). No comparable peaks were detected in the chromatograms of the two control samples (sample A, final wash eluate; sample C, vehicle desorption eluate) (Fig. 4c and d). By comparison of the retention times and UV absorption profiles of K-1 and K-2 with the HPLC fingerprint of the alkaloid extract of CB, components K-1 and K-2 were identified as peaks 2 and 3 (Fig. 4b). These findings identified the RAW 264.7 cell-binding molecules K-1 and K-2 as potential anti-inflammatory components of CB.

### Identification of K-1 and K-2 by HPLC–MS

**Fig. 5:**
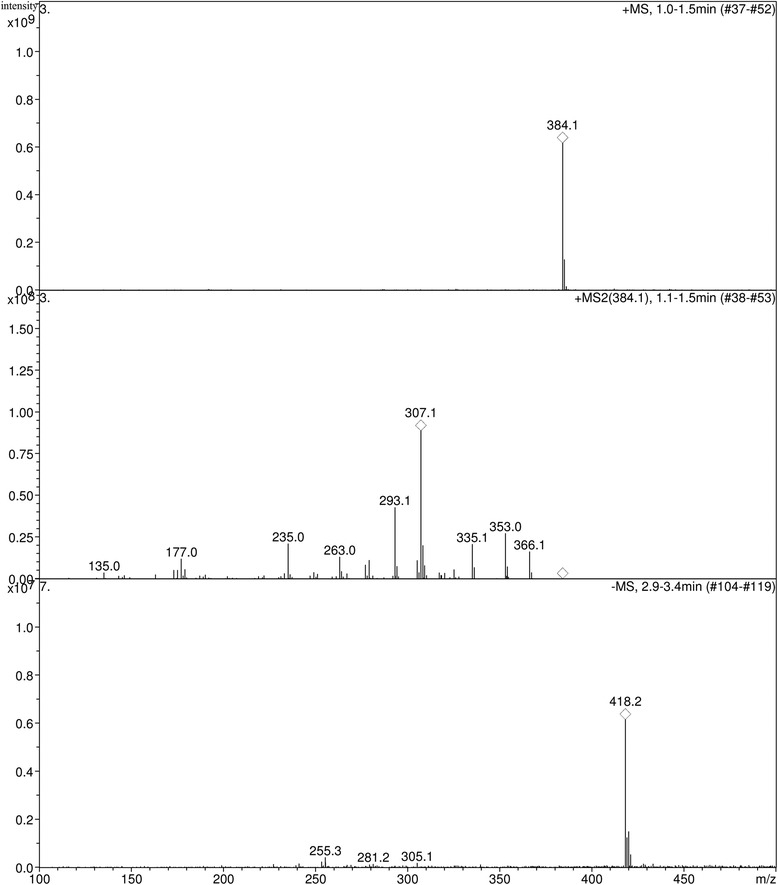
ESI-MS negative ionization spectrum of K-2 obtained from macrophage binding

5 mg of K1 and K2 were respectively dissolved in d6-CDCl_3_ as the samples, and detected by Bruker AV-500. The MS spectrum of compound K-2 is shown in Fig. 5. The mass spectrum of compound K-2 was similar to the mass spectrum of 12-hydoxycorynoline. Identification was supported further by ^1^H-NMR data and ^13^C-NMR data: ^1^HNMR (500 MHz, CDCl3): 6.661 (1H, s, 1-H), 7.023 (1H, s, 4-H), 2.177 (3H, s, 5-N-CH3), 3.437, 4.020 (2H, ABq, J = 15.5, 6-H), 6.808 (1H, d, J = 8.0, 9-H), 6.962 (1H, d, J = 8.0, 10-H), 3.878 (1H, s, 11-H), 4.902 (1H, s, 12-H), 1.242 (3H, s, 13-CH3), 3.310 (1H, s, 14-H), 5.995 ~ 5.942 (4H, m, 2-O-CH2-O-3, 7-O-CH2-O-8); ^13^C-NMR (125 MHz, CDCl3): 107.8 (C-1), 125.9 (C-1a), 145.1 (C-2), 147.0 (C-3), 112.0 (C-4), 128.8 (C-4a), 43.1 (5-N-CH3), 54.0 (C-6), 116.3 (C-6a), 142.7 (C-7), 148.6 (C-8), 109.9 (C-9), 118.9 (C-10), 135.5 (C-10a), 74.0 (C-11), 81.4 (C-12), 39.7 (C-13), 23.7 (13-CH3), 70.2 (C-14), 101.4 (2-O-CH2-O-3), 101.4 (9-O-CH2-O-10).

Compound K-1 was postulated to be corynoline. Under identical chromatographic conditions, the minor polar compounds of K-1 and the retention time of the reference peak of corynoline were identical.

### Anti-inflammatory activity of corynoline and 12-hydroxycorynoline in vitro

**Fig. 6:**
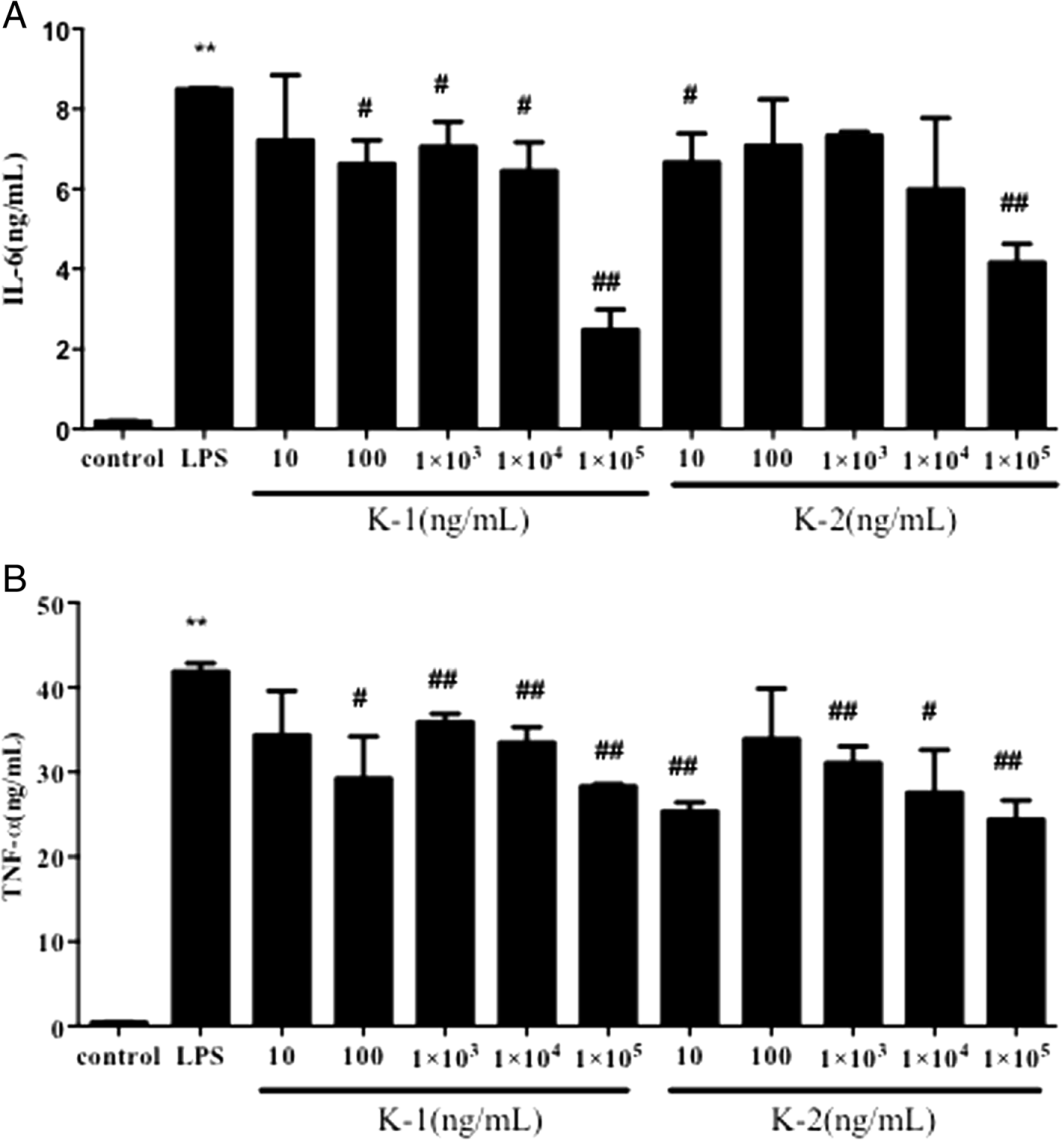
Anti-inflammatory effect of corynoline and 12-hydroxycorynoline on the level of IL-6 (**a**) a nd TNF-α (**b**) induced by LPS in RAW 264.7 cells. Data are expressed as mean ± SD (*n* = 10). *p < 0.05 and **p < 0.01 vs. model group

**Fig. 7:**
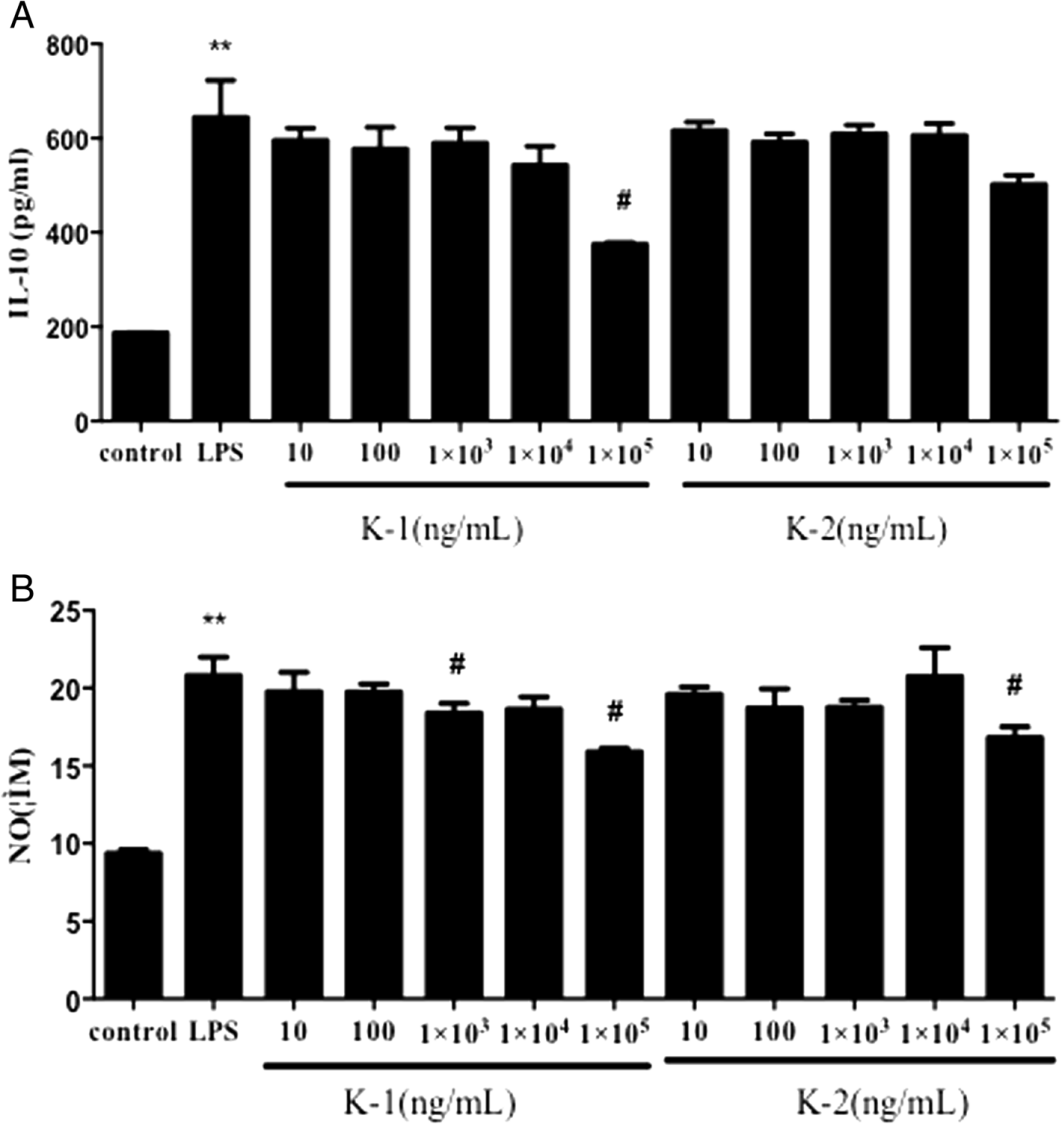
Anti-inflammatory effect of corynoline and 12-hydroxycorynoline on the level of IL-10 (**a**) and NO (**b**) induced by LPS in RAW 264.7 cells. Data are expressed as mean ± SD (*n* = 10). *p < 0.05 and **p < 0.01 vs. model group

ELISA assay was applied to examine the effect of corynoline and 12-hydroxycorynoline on IL-6, TNF-α, IL-10 and NO levels in LPS-treated cells. As showed in Figs. 6 and 7, LPS alone dramatically increased the levels of IL-6, TNF-α, IL-10 and NO. However, corynoline or 12-hydroxycorynoline at the certain concentrations obviously inhibited LPS-induced IL-6 and TNF-α overproduction^.^ (*P <* 0.01 or *P* < 0.05). Corynoline (100 μg/mL) significantly suppressed LPS-induced IL-10 overproduction (*P* < 0.05) while 12-hydroxycorynoline had no obvious effect on this index (Fig. 7a). In addtion, Corynoline (1, 100 μg/mL) and 12-hydroxycorynoline (100 μg/mL) significantly inhibited LPS-induced NO production (Fig. 7b).

## Discussion

CB, a perennial herb, has been shown to possess multiple biological activities, such as anti-bacterial and anti-viral. Its extract contains abundant alkaloids which are responsible for the potential pharmacological activity and been used as heat-clearing and detoxifying agent. However, there is little knowledge about the anti-inflammatory activity and the potential anti-inflammation components. In the present study, we used aspirin as positive anti-inflammatory compound because it is recognized as antipyretic analgesics. Compared with it, we found that the total alkaloids of CB have a significant anti-inflammatory activity in vitro and in vivo. More importantly, compounds K-1 and K-2 bound to macrophages which have been identified as 12-hydmxycorynoline and corynoline can inhibit LPS-induced inflammatory responses in RAW 264.7 cells.

The interesting and significant efficacy of herbs or TCM has aroused the increasing interest of many researchers. However, existing research is very difficult due to its complex components. The active ingredient in Chinese medicine and herbal medicine has been the focus of research. Cells membrane chromatography (CMC), a chromatographic biological affinity method that uses specific cell membranes as the stationary phase, has been confirmed as a simple, specific, effective screening method based on the binding principle that the recognition and interaction of drug molecules and targets, receptors, channels on the cell membrane [14]. A novel peritoneal macrophage/cell membrane chromatography (PM/CMC)-HPLC/MS method was shown to be an effective screening system for the rapid detection, enrichment, and identification of anti-inflammatory components from TCM [14]. Herein, we carried out this effective screening method to screen anti-inflammatory components from CB. The structure identification by spectral and chromatographic techniques provided the evidence that corynoline and 12-hydmxycorynoline are the binding components to RAW 264.7 cells. Their anti-inflammatory activities were proved in our study. This study supported the notion that the binding of RAW 264.7 cells combined with HPLC provides a rapid and efficient method for the identification of potential anti-inflammatory components in complex mixtures derived from medicinal herbs used in TCM.

Endotoxin LPS are the major molecular component of the outer membrane of Gram-negative bacteria and may excite the local inflammatory response [15]. The inhibition of LPS-induced pro-inflammatory cytokines from macrophages can be archived by LPS immobilization on porous and non-porous [16]. The stimulation of LPS could cause inflammatory response and regulate the release of inflammatory mediators. The potential inhibition of components on LPS-induced inflammatory response has been used to evaluate the anti-inflammatory activity.

Corynoline is the major alkaloid component derived from CB. Corynoline, acetylcorynoline, and protopine can significantly reduce CCl_4_-induced microsomal lipid peroxidation and attenuates 2,4-dinitro-1-fluorobenzene-induced delayed-type hypersensitivity. Some isoquinoline alkaloids such as 12-hydroxycorynoline isolated from CB hold potential bacteriostatic activity. LPS stimulates innate immunity by regulating the production of inflammatory mediators such as NO, TNF-α, IL-6, prostanoids, and leukotrienes [17]. For example, IL-6 overexpression is involved in rheumatoid arthritis [18]. TNF-α exhibits its pro-inflammatory activity by regulating several intercellular and vascular cell adhesion molecules, which results in the recruitment of leukocytes to inflammation sites [19]. In vitro bioassays have demonstrated that corynoline and 12-hydroxycorynoline in CB significantly inhibit the production of certain inflammatory mediators, especially TNF-α and IL-6. It has been suggested that corynoline and 12-hydroxycorynoline contribute to the anti-inflammatory effects of CB.

Overall, the present study demonstrated the efficacy of the alkaloid extract of CB in different anti-inflammatory tests. Our established screening system showed that 12-hydroxycorynoline and corynoline were two important active components for the inhibition of LPS-stimulated inflammatory responses. The anti-inflammatory effects of CB might be due at least in part to the corynoline and 12-hydroxycorynoline present in the plant. These two compounds might be regarded as potential candidate molecules for inflammation inhibitors.

## Conclusions

Our experimental results indicated that corynoline and 12-hydroxycorynoline contribute to the anti-inflammatory effects of the alkaloid extract of CB. Our findings also suggested that these two compounds can be used as candidate for anti-inflammatory drugs.
